# Nanoclay- and TiO_2_ Nanoparticle-Modified Poly(*N*-vinyl pyrrolidone) Hydrogels: A Multifunctional Material for Application in Photocatalytic Degradation and Adsorption-Based Removal of Organic Contaminants

**DOI:** 10.1021/acsomega.2c04595

**Published:** 2022-09-22

**Authors:** Salsabil Marouch, Noura Benbellat, Ali Duran, Erkan Yilmaz

**Affiliations:** † Laboratory of Chemistry and Environmental Chemistry (LCCE), Department of Chemistry, Faculty of Matter Sciences, Batna-1 University, 05000 Batna, Algeria; ‡ Department of Analytical Chemistry, Faculty of Pharmacy, Erciyes University, 38039 Kayseri, Turkey; § Nanotechnology Application and Research Center, ERNAM Erciyes University, 38039, Kayseri, Turkey; ∥ Laboratory of Chemistry of Materials and Living: Activity & Reactivity (LCMVAR), Department of Chemistry, Faculty of Matter Sciences, Batna-1 University, 05000 Batna, Algeria; ⊥ Department of Nanotechnology Engineering, Faculty of Engineering, Abdullah Gul University, 38080 Kayseri, Turkey; # Technology Research and Application Center (TAUM), Erciyes University, 38039 Kayseri, Turkey

## Abstract

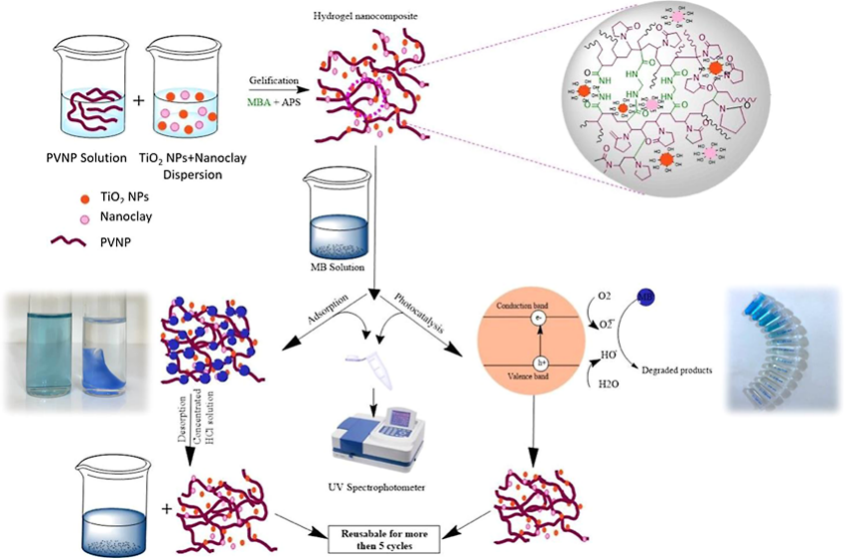

In recent times, access to clean water has become increasingly difficult and one of the most important problems for the sustainability of life due to environmental pollution. Based on this thought, in this study, a multifunctional hydrogel nanocomposite (nanoclay@TiO_2_@PNVP) containing linear poly(*N*-vinyl pyrrolidone) (PNVP), nanoclay, and TiO_2_ nanoparticles was synthesized and used as an adsorbent and photocatalyst for the adsorption-based and photocatalytic degradation-based removal of organic and pharmaceutical pollutants such as methylene blue (MB) and sildenafil citrate (SLD). The modification of the hydrogel with TiO_2_ nanoparticles and nanoclay aimed to increase the adsorption capacity of the PNVP hydrogel as well as to gain photocatalytic properties for the effective removal of organic contaminants. This hybrid material, which can be cleaned in two different ways, can be reused and recycled at least 10 times. Characterization studies were carried out using Fourier transform infrared spectroscopy, scanning electron microscopy, Raman spectroscopy, thermogravimetric analysis, differential thermogravimetry, and viscosimetry techniques. Optimization studies for the adsorption-based removal of organic contaminants were carried out on MB and SLD as model organic compounds. The optimum parameters for MB were found at pH 10 of the sample solution when 50 mg of the nanoclay@TiO_2_@PNVP hydrogel nanocomposite was used for 420 min of contact time. It was observed that 99% of the MB was photocatalytically degraded within 150 min at pH 10. Our material had multifunctional applicability properties, showing high adsorption and photocatalytic performances over 99% for at least 10 times of use. For the removal of organic and pharmaceutical contaminants from wastewater, the synthesized material can be used in two treatment processes separately or in combination in one step, providing an important advantage for its usability in environmental applications.

## 1 Introduction

Water pollution is a huge environmental problem, global water consumption is increasing with the growth of the human population and about 780 million people around the world cannot afford pure water for drinking.^1,2^ To overcome this problem, many chemical and physical approaches have been developed, such as chemical precipitation, membrane filtration, coagulation, adsorption, chemical oxidation, and photocatalysis, each of these methods has its advantages and disadvantages, some of them are expensive and some are not effective enough.^3^ However, among these techniques, adsorption, and photocatalytic degradation appear to offer the best prospects over all the other methods. They are effective, simple, green, and promoting techniques since the used materials can be recycled and reutilized several times, making the cost of the dye removal reasonable.

Adsorption is a separation method in which a solid material (adsorbent) can form electrostatic bonds through functional groups on its surface and selectively remove dissolved contaminants from a solution.^4,5^ Many adsorbents have been used in this regard such as activated carbons which, despite their cost, are the most commonly used adsorbents.^6^ Clays also received a big attention thanks to their low cost, nontoxicity, availability, potential ion exchange, and adsorption properties. Especially, nano clays exhibit high surface area and surface reactivity making them good adsorbents for organic and inorganic contaminants.^7^

In recent years, extensive studies have been conducted on the photocatalytic technique since it can be used for pollutant degradation, hydrogen energy production, and energy conversion.^8^ Classified as a green technique, photocatalysis is mainly based on converting photonic energy to chemical energy by the intermediate of some semiconductors (photocatalysts).^9−11^ During this process, a series of oxidation–reduction reactions are involved in which water or oxygen molecules are activated by photogenerated charge carriers to generate hydroxyl radicals (^•^OH) and superoxide radicals (^•^O_2_^–^). These active spices are highly oxidative and reactive; entering in reaction with the harmful molecule, they provoke its degradation into harmless small molecules or CO_2_ and H_2_O.^12,13^

Many nanomaterials have been used as photocatalysts.^14−17^ Among them TiO_2_, thanks to its excellent photocatalytic performances under ultraviolet (UV) irradiation, chemical stability, long durability, nontoxicity, and low cost^18−21^ is the most widely used photocatalyst.

Nanomaterials demonstrated their immense capability and potential for water purification, but their small size makes their separation from the treated water hard and expensive since they stay suspended in water, penetrate through filtration materials, and clog their pores.^2^ To facilitate the separation process, nanoparticles have been entrapped in different materials such as polymer films, aerogels, and hydrogels. The use of such supports helps not only to facilitate the separation but also helps to enhance the agglomeration of the nanoparticles which results in a reduction of their potential activity.^22,23^

Hydrogels are three-dimensional networks of polymers formed by chemical or physical crosslinking of hydrophilic polymer chains. They are characterized by their ability to absorb large amounts of water and organic solvents without dissolving or losing their structures.^24^ A hydrogel is a name given to a network in which the solvent content must be greater than 20%; if this exceeds 95%, the hydrogel is called super absorbent. This characteristic of high absorption comes from the hydrophilic groups which constitute the polymer backbone such as hydroxides (−OH−), carboxyls (−COOH−), amides (−CONH– or −CONH_2_−), or sulfonic (−SO_3_H−), and thanks to these groups, hydrogels receive a huge interest in water purification domain since they can attract and adsorb various pollutants such as heavy metals and organic dyes. Hydrogels can be used as pure or hybrid forms^25^ associated with specific nanoparticles to enhance the properties of the network such as mechanical,^26^ adsorption, and photocatalytic properties.^27^ The pores present in the structure of hydrogels serve as a transport pathway allowing the permeation of wastewater into the hydrogel leading to a better contact surface area between the dye, hydrogel, and nanoparticles.^26,28−30^

Numerous studies have been reported on the application of multifunctional materials for water purification.^31−33^ These materials present the advantage of using the same material for one process separately (adsorption or photocatalysis) or by combining the two processes in one step process, which simplifies the treatment process and leads to a decrease in the technological cost since, after adsorption, there is no need to a postfiltration to recycle the material, and the dye is directly degraded under UV light.

In this study, a multifunctional poly(*N*-vinyl pyrrolidone) (PNVP) hydrogel including TiO_2_ NPs and nanoclays was synthesized and utilized for the removal of methylene blue (MB) and sildenafil citrate (SLD) from wastewater by both adsorption and photocatalytic degradation mechanisms. By modifying the hydrogel with TiO_2_ NPs and nanoclays, both adsorption and photocatalytic properties were gained.

## 2 Experimental Section

### 2.1 Chemicals and Reagents

*N*-Vinyl pyrrolidone (NVP 99%), 2,2′-azobis(2-methyl propionamidine) dihydrochloride (AAPH 99%), *N*,*N*′-methylene-bis-acrylamide (MBA 99%), ammonium persulfate (APS 98%), and titanium dioxide nanoparticles (TiO_2_) (particle size 21 nm) were all purchased from Sigma-Aldrich. Nanoclay (particle size 30 nm) was obtained from Nanografi Nano Technology. Deionized water (resistivity 18.2 MΩ cm) was obtained using Milli-Q system deionized water system (Millipore, USA). All chemicals and solvents were used as received without further purification.

### 2.2 Synthesis of the Linear PNVP

Hydrogel and hydrogel nanocomposites were prepared in plastic bottles by mixing components of materials. The PNVP was prepared by free radical solution polymerization using AAPH as a water-soluble thermal initiator. NVP (0.089 mol) and AAPH (0.83 × 10^–3^ mol) were dissolved in 100 mL of deionized water; then, oxygen was chased from the solution using nitrogen bubbling. The solution was then placed in a 55 °C bath and stirred for 12 h. The formed PNVP was precipitated using acetone and washed several times by dissolution/precipitation process using water and acetone, respectively. The pure PNVP was left to dry at room temperature and then placed in an oven at 50 °C for 24 h.

### 2.3 Synthesis of Hydrogel and Hydrogel Nanocomposites

**Figure 1:**
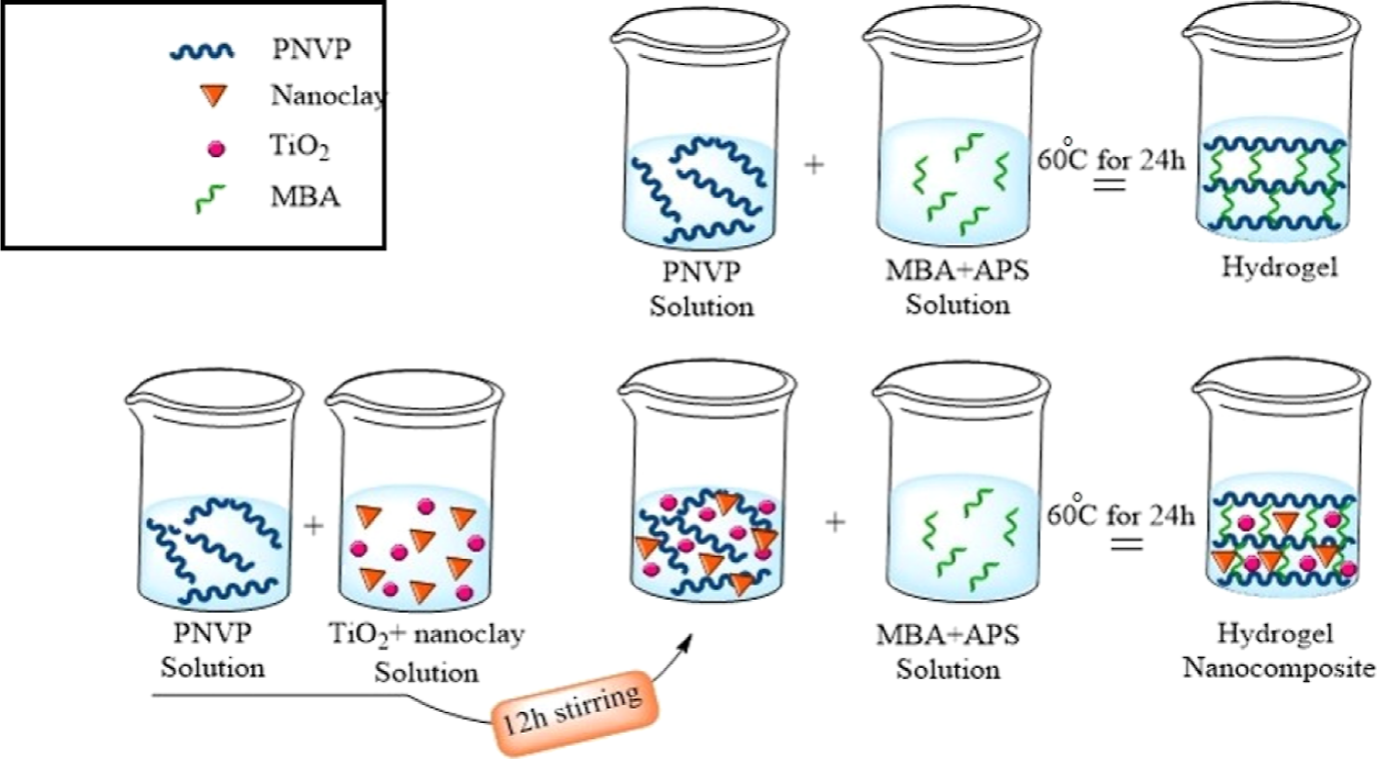
Schematic diagram showing the experimental steps in the preparation of hydrogel and hydrogel nanocomposites.

2 g of PNVP were dissolved in 10 mL of water (20%); at the same time, solutions including different proportions of TiO_2_ NPs and nanoclays were prepared (Table 1). The prepared solutions including nanomaterials were placed in an ultrasonic bath for 1 h; then, the dispersed nanoparticles were slowly added to the prepared polymer solutions. The mixtures obtained were sonicated for 1 h and then placed under stirring for 12 h; after that, jellification of the polymer solutions occurred by adding a solution of 5 mL of a mixture of APS (40%) and MBA (4%), respectively. The quantities of each of TiO_2_, nanoclay, APS, and MBA were taken based on polymer weight. After homogenization, the solutions were placed in an oven under 60 °C for 5 h. The formed hydrogels were washed with distilled water until constant weight. The sequence of preparation of the hydrogel and hydrogel nanocomposites is shown in Figure 1.

**Table 1:** Formulations of Hydrogel and Nanocomposite Hydrogels

sample	PNVP (g)	TiO_2_ (%)	nanoclay (%)	APS (%)	MBA (%)
PNVP hydrogel	2			40	4
TiO_2_@PNVP	2	4		40	4
nanoclay@PNVP	2		4	40	4
nanoclay@TiO_2_@PNVP	2	4	4	40	4

### 2.4 Instrumentation

The chemical composition of the synthesized PNVP, PNVP hydrogel, TiO_2_@PNVP, nanoclay@PNVP, and nanoclay@TiO_2_@PNVP hydrogel nanocomposites was characterized by Fourier transform infrared spectroscopy (FT-IR, Thermo Scientific Nicolet 6700 spectrometer, using attenuated total reflectance method) and Raman spectroscopy (alpha300 Raman spectrometer, equipped with a 532 nm laser source) techniques. The morphology was studied using the ZEISS EVO LS10 scanning electron microscopy (SEM) at an operating voltage of 10 kV, and thermal behavior and stability studies were evaluated using thermogravimetric analysis (TGA) and differential thermogravimetry (DTG) [PerkinElmer Diamond DTA–TGA thermal analyzer. The samples (2.18–6.71 mg) were placed in a platinum pan and heated up to 800 °C at a rate of 10 °C/min under nitrogen purge]. The viscosimetric average molecular weight of the linear PNVP was determined using an Ubbelohde capillary viscometer. The final photodegraded intermediate was identified by gas chromatography-tandem mass spectrometry (GC–MS). The amounts of MB and SLD were determined by a UV–vis spectrophotometer and a ultra-performance liquid chromatography (UPLC) system.

#### 2.4.1 Determination of the Molecular Weight of PNVP by Intrinsic Viscosity

The viscosimetric average molecular weight of PNVP was determined in water, using an Ubbelohde capillary viscometer at 30 ± 0.1 °C. Flow times of water (*t*_s_) and five different concentrations of PNVP solution (*t*) were recorded, and the following viscosities were calculated according to the given equations (eqs 1–4)
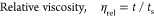
1
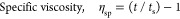
2
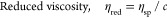
3
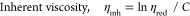
4

The value of intrinsic viscosity [η] is determined by extrapolation to zero concentration of the graph representing the inherent viscosity as a function of concentration, and the intrinsic molecular weight (*M*) is directly related to the intrinsic viscosity [η] according to the Mark–Houwink–Sakurada equation (eq 5).
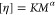
5where *K* and α are characteristic constants for a given polymer/solvent couple at a given temperature.

### 2.5 Swelling of the Hydrogel and Hydrogel Nanocomposites

The percentage of swelling of the synthesized materials was evaluated through water uptake as a function of time by the gravimetric method. 50 mg of each sample was immersed in 20 mL of deionized water at room temperature; then, the mass of the swollen materials was evaluated at different intervals of time until equilibrium swelling was attended. Water uptake was calculated according to the following equation (eq 6)
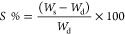
6where *W*_s_ is the weight of the swollen sample at time *t*, and *W*_d_ is the weight of the dried hydrogel sample.

### 2.6 Adsorption-Based Removal Experiments

To evaluate the adsorption performance of MB, adsorption experiments were carried out 3 times under the same conditions at room temperature for different pH values ranging from 3 to 10. In the experiment, 50 mg of each material was immersed into 20 mL of MB solution (2 mg·L^–1^) in which the initial pH was adjusted by a buffer solution. Within 24 h contact time, a volume of 1 mL was taken from the sample solution, and the amounts of residual MB were determined by a UV–vis spectrophotometer at λ_max_ = 664 nm.

The adsorption percentage is given by the following equation (eq 7)
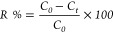
7where *C*_0_ (mg·L^–1^) and *C*_*t*_ (mg·L^–1^) are the MB concentration at the initial time and time *t*, respectively.

The effect of contact time on the adsorption-based removal of MB was evaluated using the previous experiment at pH 10. A volume of 1 mL was taken from sample solutions at different contact times until equilibrium was reached. The solution is then analyzed with UV–vis to determine the amounts of residual MB.

Adsorption isotherm of the optimized material at pH 10 was studied by immersing 50 mg of the hydrogel-based nanomaterial in 20 mL of MB solution of various concentrations (2–100 mg·L^–1^).

The adsorption capacity of the material was calculated using the following equation (eq 8)
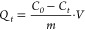
8where *q*_e_ (mg·g^–1^) is the equilibrium adsorption capacity, *C*_0_ (mg·L^–1^) and *C*_e_ (mg·L^–1^) are the initial and equilibrium concentration of MB, respectively, *V* (L) is the volume of MB solution, and *m* (g) is the mass of the adsorbent.

The adsorption mechanism of MB by the synthesized materials was explored according to the pseudo-first-order, pseudo-second-order, and intraparticle diffusion models using the following equations (eqs 9–11).
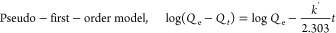
9
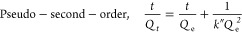
10
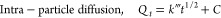
11where *Q*_e_ (mg·g^–1^) represents the maximum adsorption capacity; *Q*_*t*_ (mg·g^–1^) represents the adsorption capacity at time *t* (min); *k*′ (mg·g^–1^ min^–1^), *k*″ (mg·g^–1^ min^–1^), and *k*‴ are the constant rates of the pseudo-first-order, the pseudo-second-order, and the intraparticle diffusion models, respectively, and *C* (mg·g^–1^) means the thickness of the boundary layer.^34^

The optimized material was used for adsorption-based removal of SLD. In the experiments, 50 mg of nanoclay@TiO_2_@PNVP hydrogel nanocomposites was immersed for 24 h in 20 mL of 2 mg·L^–1^ SLD solution at different pH values ranging from 2 to 10.

The photodegradation of SLD was also studied using the optimized nanoclay@TiO_2_@PNVP hydrogel nanocomposite at different pH values. UPLC-diode array detector was used to determine the concentration of SLD before and after the adsorption and photocatalysis.

### 2.7 Photocatalytic Degradation Experiments

The photodegradation experiments of MB using PNVP hydrogel, TiO_2_@PNVP, nanoclay@PNVP, and nanoclay@TiO_2_@PNVP hydrogel nanocomposites were conducted using 150 mL of MB solution (20 mg·L^–1^) in which 0.25 g of each material was immersed. The mixture was stirred in a dark chamber until the equilibrium of adsorption was achieved, transferred to the photocatalytic reactor, and subjected to 400 W of a UV halogen lamp. The rate of photodegradation is evaluated every 30 min, a volume of 1 mL was piped from the residual MB solution and analyzed using a UV–vis spectrophotometer. The degradation percentage was calculated using the following equation (eq 12)
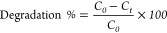
12The pH effect on MB photodegradation is also studied using the nanoclay@TiO_2_@PNVP hydrogel nanocomposite material at different pH values of MB solutions ranging from pH 2 to 10.

The rate and kinetics of MB removal are explored using zero-order, pseudo-first-order, and pseudo-second-order kinetic models (eqs 13–15).
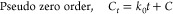
13
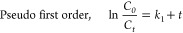
14
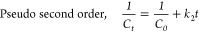
15where *C*_0_ is the initial concentration of MB (mg·L^–1^) and *C*_*t*_ is the concentration of MB at time *t* (mg·L^–1^), *t* is the contact time in hours, and *k*_0_, *k*_1_, and *k*_2_ are zero-order, first-order, and second-order rate constants, respectively.^35^

To understand the mechanism of photodegradation, photocatalytic experiments were performed in presence of 1 M of isopropyl alcohol, CuCl_2_, benzoquinone, and 0.2 M of EDTA separately to entrap the radicals involved in the process.

The reusability of the synthesized nanoclay@TiO_2_@PNVP hydrogel nanocomposite was assessed 10 times for photocatalysis and 5 times for adsorption-based removal of MB. After each cycle, the sample was filtered, and the hydrogel nanocomposite was put in 250 mL of 2 M HCl solution for MB desorption and then washed 2 times with distilled water.

## 3 Results and Discussion

### 3.1 Synthesis of PNVP, PNVP Based Hydrogel, and Hydrogel Nanocomposites

In this study, PNVP chains were prepared by free radical solution polymerization using AAPH as a thermal initiator, the use of this initiator permitted to conquer the secondary reactions that may occur during NVP polymerization.^36^ What results in a high conversion rate of 96%.

PNVP hydrogel and hydrogel nanocomposites were synthesized by crosslinking of PNVP in presence of APS and MBA. Under heat effect, radicals formed by the decomposition of APS tear-off radical hydrogens from the tertiary carbon of the polymer chains, allowing a polymer chain or MBA to get attached. The succession of this reaction leads to the formation of a network. The gelation percentages were calculated using an equation (eq 16) and they ranged from 96 to 99% (Table 2)
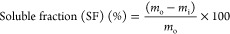
16
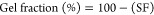
17where (*m*_o_) is the initial weight of the dry hydrogel before purification and (*m*_i_) is the weight of the dry hydrogel after purification.

**Table 2:** Percentages of Soluble and Gel Fractions of Different Samples of PNVP-Based Hydrogels

sample	soluble fraction (%)	gel fraction (%)
PNVP hydrogel	3.66	96.34
TiO_2_@PNVP hydrogel	2.56	97.44
nanoclay@PNVP hydrogel	1.03	98.97
nanoclay@TiO_2_@PNVP hydrogel	1.09	98.91

### 3.2 Characterization of the Synthesized Materials

The molecular weight of PNVP plays an important role in the gelation and the properties of the hydrogel networks, so it is important to determine the molecular weight of the synthesized polymer. For this purpose, the intrinsic viscosity method was realized to evaluate the molecular weight of the synthesized PNVP. Mark–Houwink–Sakurada coefficients were taken from literature, *K* = 1.4 × 10^–4^ and α = 0.7,^37^ and the molecular weight of PNVP was found to be *M* = 407 532.551 g·moL^–1^.

**Figure 2:**
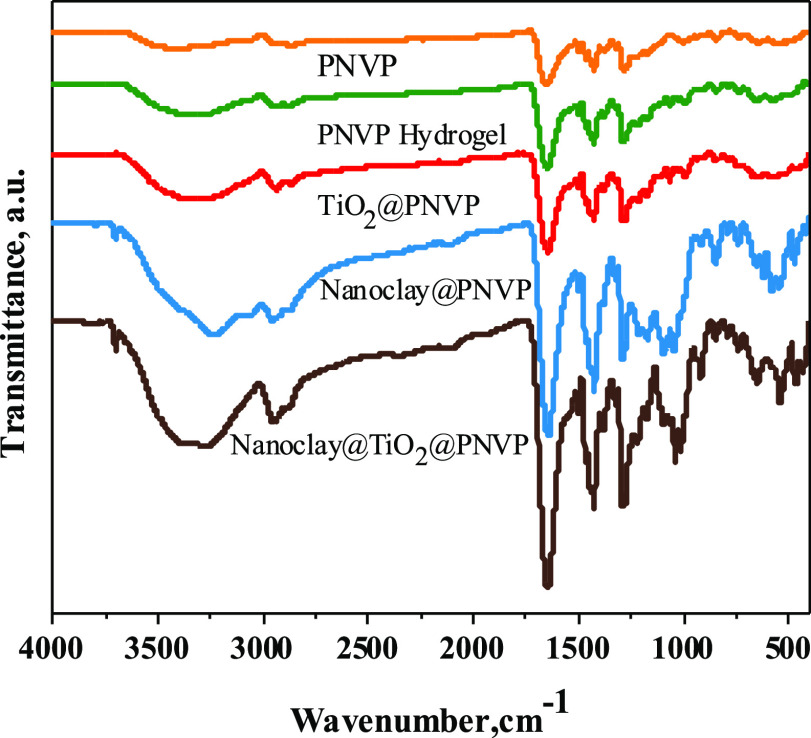
FT-IR spectra of PNVP, PNVP hydrogel, TiO_2_@PNVP, nanoclay@PNVP, and nanoclay@TiO_2_@PNVP hydrogel nanocomposites.

FT-IR spectra of dried PNVP, PNVP hydrogel, TiO_2_@PNVP, nanoclay@PNVP, and nanoclay@TiO_2_@PNVP hydrogel nanocomposites are presented in Figure 2. For all samples, the characteristic peaks of PNVP located around 985, 1286, 1462, and 1644 cm^–1^ were associated with C–N and C=O stretching vibrations. Moreover, bands at 1316 and 1420 cm^–1^ were assigned to the deformation of CH and CH_2_ bounds, respectively.^38^ The TiO_2_ NPs modified PNVP hydrogels showed additional new peaks around 554 and 655 cm^–1^ which can be attributed to TiO_2_ NPs. For nanoclay-based PNVP hydrogels, bands around 3588, 1032, and 912 cm^–1^ were observed, and these peaks were assigned to hydroxyl stretching of Al–OH and Si–OH, Si–O bond stretching vibrations, and Al_2_OH deformation vibration, respectively.^39^ The sharpness of the absorption bands remained at almost the same frequencies, and slight changes were observed in intensities. This means that there was no formation of new functional groups and that the gelation did not affect the structure of PNVP since the network was formed by C–C bonds.

**Figure 3:**
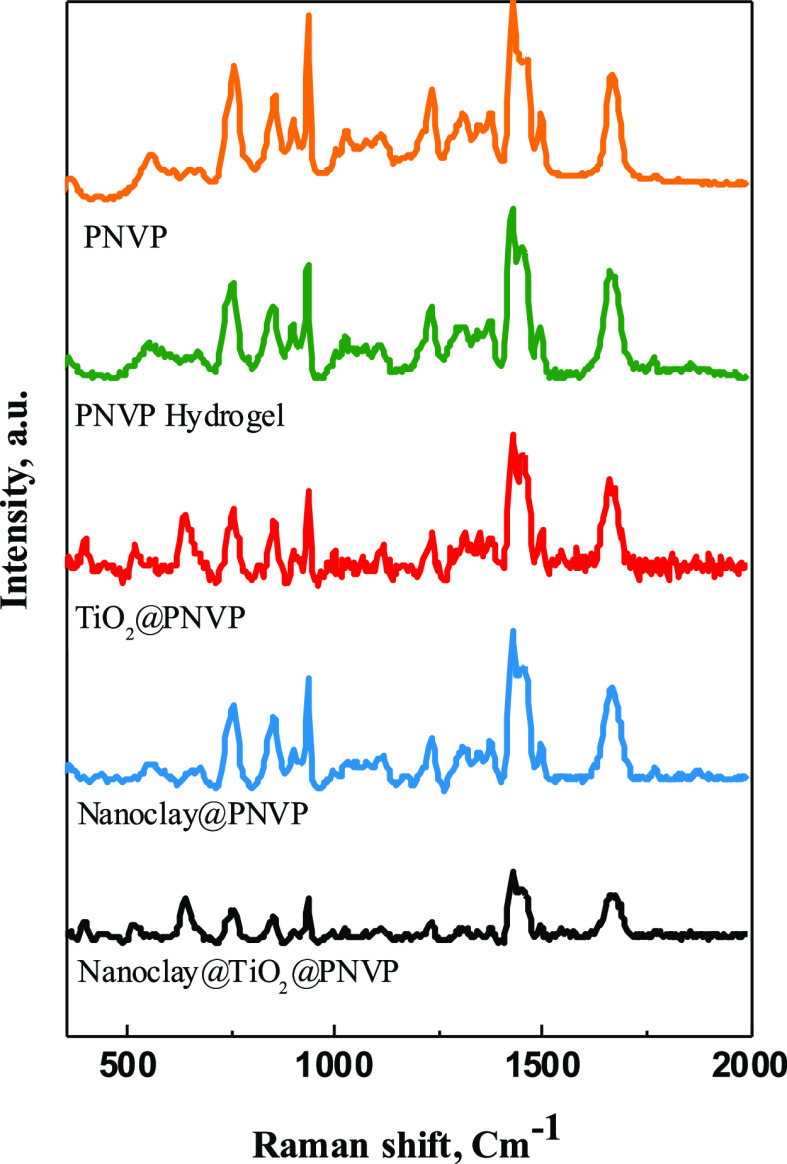
Raman spectra of PNVP, PNVP hydrogel, TiO_2_@PNVP, nanoclay@PNVP, and nanoclay@TiO_2_@PNVP hydrogel nanocomposites.

The prepared materials were further characterized using Raman spectroscopy techniques. Figure 3 displays the Raman spectra of dried PNVP, PNVP hydrogel, TiO_2_@PNVP, nanoclay@PNVP, and nanoclay@TiO_2_@PNVP hydrogel nanocomposites. For all samples, the presence of PNVP was confirmed by the appearance of the characteristic peaks at 937, 1232, 1460, and 1670 cm^–1^ which were assigned to C–C ring breathing mode, C–N, N–C=O (symmetric), and C=O stretching vibrations, respectively. Besides, peaks at 1306 and 1427 cm^–1^ were associated with the deformation of CH and CH_2_ bounds, respectively.^40^ Characteristic peaks at 516 and 638 cm^–1^ were observed for TiO_2_ NP-based PNVP hydrogel nanocomposites.^41^ For nanoclay-based materials, peaks around 1002 and 440 cm^–1^ were observed, and they were related to Si–O vibrations.^42^

**Figure 4:**
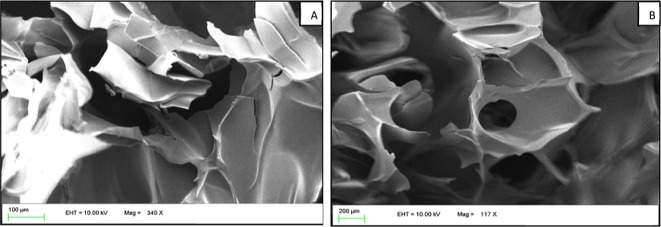
Scanning electron microscopy analysis of the PNVP hydrogel (A) and the nanoclay@TiO_2_@PNVP hydrogel nanocomposite (B).

SEM images of the PNVP hydrogel and the anoclay@TiO_2_@PNVP hydrogel nanocomposite are given in Figure 4. The PNVP hydrogel presented a soft irregular structure with some large pores, while nanoclay@TiO_2_@PNVP hydrogel nanocomposite had a tight structure with a large number of smaller-sized pores. The incorporation of nanoparticles decreased the pore dimension. This can be explained by the physical interactions that take place between the nanoparticles and the polymer matrix, and these interactions play a role in the crosslinking which leads to a denser crosslinked structure. The pore size of the optimized nanoclay@TiO_2_@PNVP hydrogel nanocomposite ranges from 300 to 400 μm like, as shown in Figure 4.

**Figure 5:**
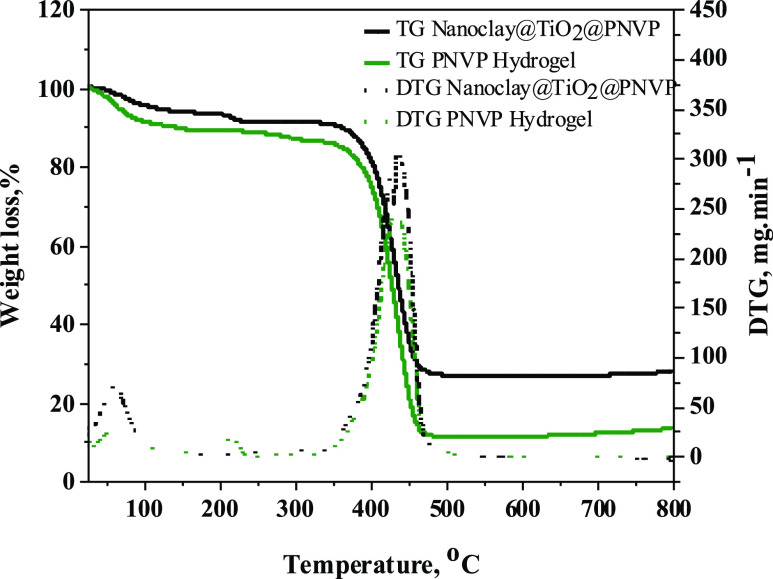
TGA and DTG of the PNVP hydrogel and the nanoclay@TiO_2_@PNVP hydrogel nanocomposite.

TGA and DTA were carried out to evaluate the thermal behavior of the synthesized materials. Figure 5 shows TGA and DTA curves of the dried PNVP hydrogel and nanoclay@TiO_2_@PNVP hydrogel nanocomposite. According to the TGA thermogram of the two samples, the weight loss takes place in two distinct steps with a major degradation step which occurs between 374 and 472 °C. The first weight loss near 100 °C was due to the evaporation of water molecules, and the second one was due to the degradation of the network. DTG curves displayed a significant decomposition peak which was observed approximately at 437 °C. The difference was seen in weight loss, and the nanoclay@TiO_2_@PNVP hydrogel nanocomposite remained more resistant and steadier within a large scale of temperature with 72% weight loss, while for PNVP hydrogel, 87% of weight loss was observed.

### 3.3 Swelling of the Synthesized Materials

**Figure 6:**
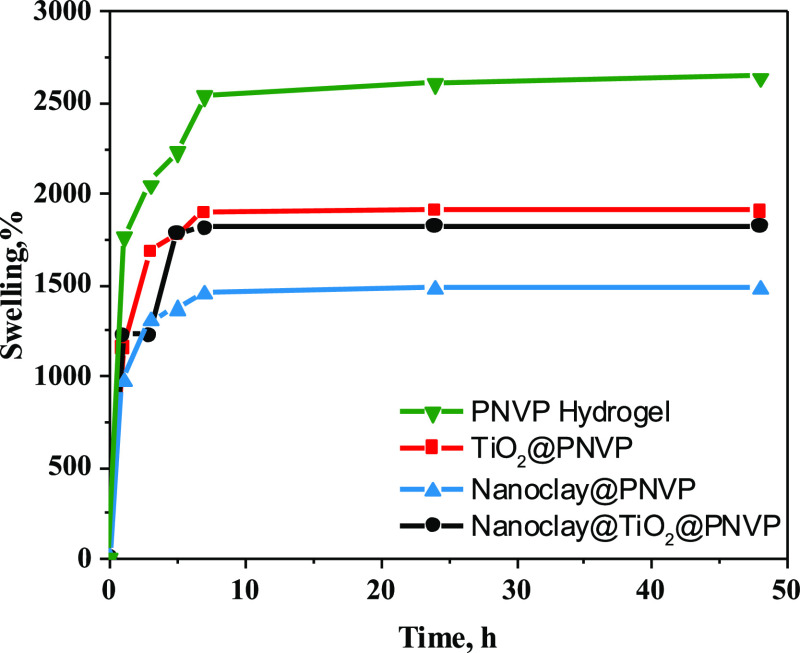
Water uptake of PNVP hydrogel, TiO_2_@PNVP, nanoclay@PNVP, and nanoclay@TiO_2_@PNVP hydrogel nanocomposites.

The swelling of the synthesized materials in water is represented in Figure 6. After 300 min of soaking, the equilibrium swelling was reached, and the maximum swelling capacity of the PNVP hydrogel was 2639%. The addition of TiO_2_ or/and nanoclay nanoparticles decreased the swelling capacity of the material by 1480% for the nanoclay@PNVP hydrogel nanocomposite. This decrease was attributed to the good interaction between the PNVP matrix and the nanoparticles. This may create physical crosslinking as was reported in the literature^43^ and supported by SEM images. The incorporation of nanoparticles leads to a denser crosslinked structure that prevents water penetration and results in a reduction of swelling ratio.

### 3.4 Adsorption-Based Removal of MB

#### 3.4.1 Effect of pH on the Adsorption-Based Removal of MB

**Figure 7:**
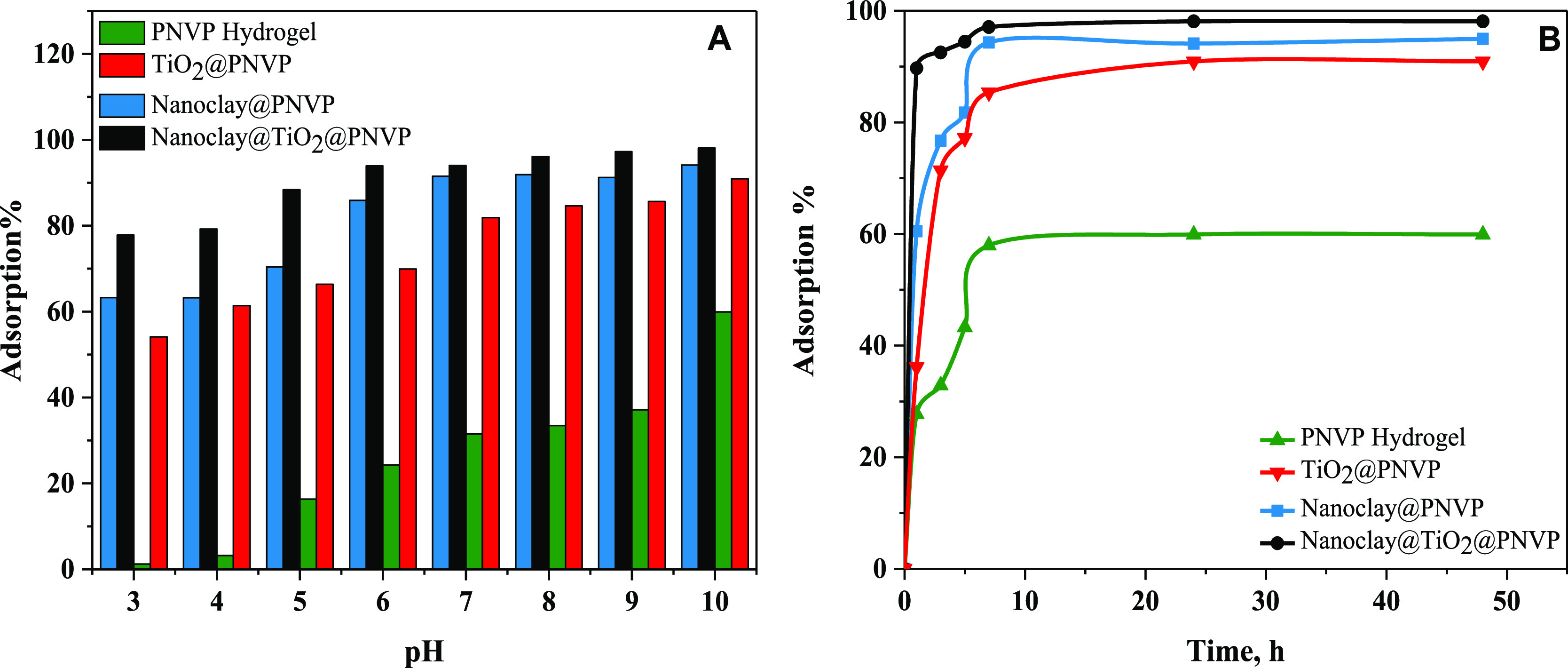
Adsorption of MB as a function of pH variation (A); adsorption of MB as a function of time (B) (*N* = 3).

To evaluate the adsorption performance of the synthesized materials, MB was used as a representative model of organic pollutants. Figure 7A represents the variation of MB adsorption percentage as a function of pH. At pH 3.0, low adsorption percentages were observed for PNVP hydrogel, TiO_2_@PNVP, nanoclay@PNVP, and nanoclay@TiO_2_@PNVP hydrogel nanocomposites. As the pH of the sample’s solution increase to pH 10, the adsorption performance increases, where the highest performances were 60, 91, 95, and 98% for PNVP hydrogel, TiO_2_@PNVP, nanoclay@PNVP, nanoclay@TiO_2_@PNVP hydrogel, respectively. These variations were associated with the electrostatic interactions between MB and the adsorbent. MB is present in a cationic form in the solutions, so the acidity of the medium plays an important role in its sequestration. In acidic mediums, the interaction of the synthesized materials with MB is limited by the protonation of the functional groups present on both the matrix and the surface of the nanomaterials such as amide and hydroxyl groups, which leads to electrostatic repulsions and lower adsorption performances. On the other hand, when pH increases, the H^+^ concentration declines and deprotonation of active functional groups occurs, so the developed materials act as nucleophiles, and they attract MB by electrostatic interactions, such as hydrogen bonding, leading to higher adsorption performances.

#### 3.4.2 Effect of Contact Time on the Adsorption-Based Removal of MB

During the adsorption process, MB continuously accumulated onto the adsorbent until it is fully saturated. To evaluate the time needed for the maximum uptake of MB, the pH of the solution was fixed at pH 10, and the adsorption percentage was calculated within different contact times (Figure 7B). The maximum adsorption of MB was attended within 7 h of immersion time. The PNVP hydrogel exhibited the lowest adsorption capacity due to low interaction with MB, and since only amide functional groups were present, TiO_2_ and nanoclay-based hydrogel nanocomposites in MB adsorption showed high adsorption capacity due to the presence of hydroxy and Si–OH functional groups, as well as amide groups.

#### 3.4.3 Adsorption Isotherm of MB by the Nanoclay@TiO_2_@PNVP

**Figure 8:**
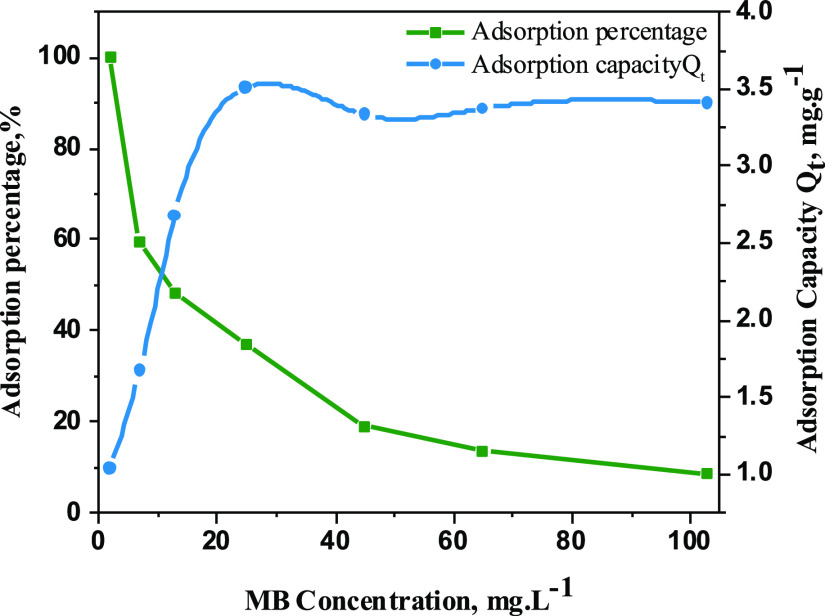
Adsorption isotherm of MB as a function of nanoclay@TiO_2_@PNVP adsorption capacity and adsorption percentage.

**Figure 9:**
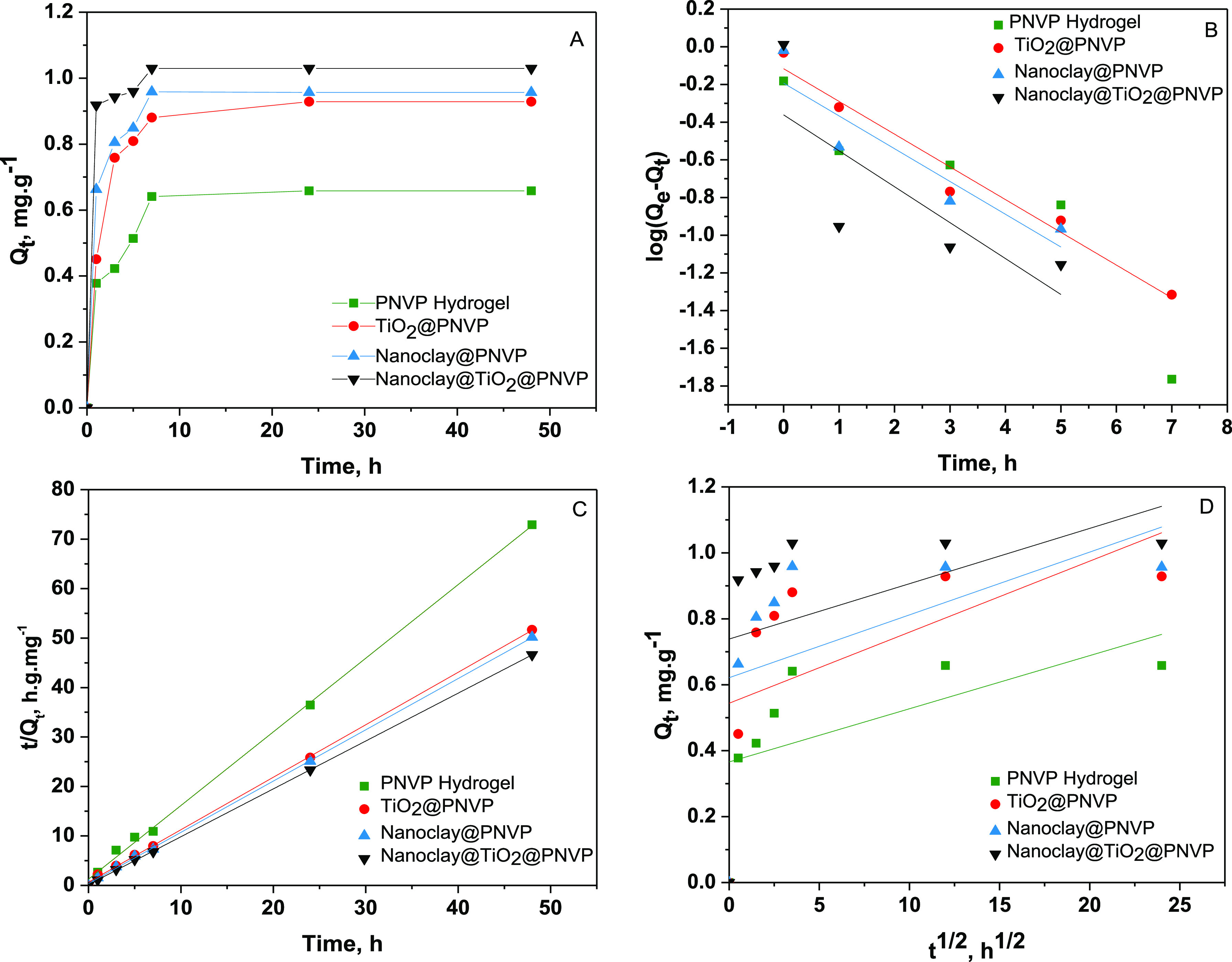
The adsorption capacity curves of the different synthesized materials (A) and the fitting curves of the pseudo-first-order kinetic model (B), pseudo-second-order kinetic model (C), and intraparticle diffusion model (D).

Adsorption capacity and adsorption percentage of nanoclay@TiO_2_@PNVP with different initial concentrations of MB were studied and given in Figure 8. The results show that the adsorption capacity at the equilibrium increases with the increase of MB concentration and does not change after it reaches 3.3 mg·g^–1^. On the other hand, the adsorption percentage is higher at lower concentrations, which means that the adsorption sites of the material are fully occupied by MB after a concentration of 20 mg·L^–1^ (Figure 9).

#### 3.4.4 Kinetics of Adsorption of MB

The kinetic parameters of the systems are as given in Table 3, based on the analysis of correlation coefficients (*R*^2^) presented in Table 3, all the synthesized materials had a better fit to the pseudo-second-order kinetic model, so the mechanism involved in MB adsorption is the chemisorption. The interactions that may exist during the adsorption process are the electrostatic interaction and hydrogen bonding, which is probably carried out by carboxylic/carboxylate, amide, and −OH groups, and by comparing the adsorption kinetics of the synthesized nanocomposite materials, the nanoclay@TiO_2_@PNVP showed the fastest kinetics with a constant rate of *k*_1_^″^ = 6.2, then comes the nanoclay@PNVP (*k*_2_^″^ = 3), TiO_2_@PNVP (*k*_3_^″^ = 1.78) and the PNVP hydrogel (*k*_4_^″^ = 1.77) respectively.

**Table 3:** Adsorption Kinetics Parameters of MB

	pseudo-first-order			pseudo-second-order			intraparticle diffusion		
sample	*Q*_e_ (mg·g^–1^)	*k*′ (mg·g^–1^ min^–1^)	*R*^2^	*Q*_e_ (mg·g^–1^)	*k*″ (mg·g^–1^ min^–1^)	*R*^2^	*C* (mg·g^–1^)	*k*‴ (mg·g^–1^ min^–1^)	*R*^2^
PNVP hydrogel	0.621	1 × 10^–3^	0.98	0.672	1.77	0.99	0.367	0.016	0.366
TiO_2_@PNVP	0.670	2 × 10^–3^	0.96	0.942	1.78	0.99	0.544	0.022	0.30
nanoclay@PNVP	0.669	4 × 10^–3^	0.78	0.965	3	0.99	0.6621	0.019	0.23
nanoclay@TiO_2_@PNVP	0.644	8 × 10^–3^	0.41	1.033	6.2	0.99	0.738	0.016	0.15

### 3.5 Photocatalytic Degradation-Based Removal MB

**Figure 10:**
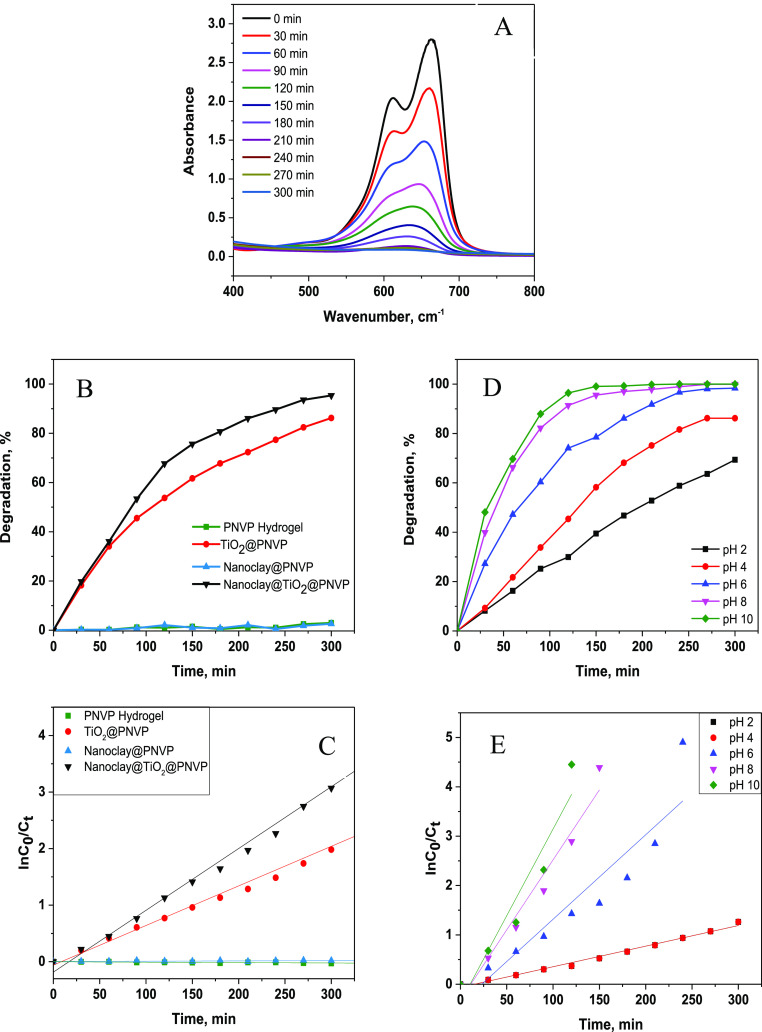
Absorbance spectrum changes of MB with photocatalytic degradation on the nanoclay@TiO_2_@PNVP hydrogel nanocomposite as a function of wavelength and UV exposition time (A), degradation of MB by the synthesized materials as a function of irradiation time (B), plot of ln(*C*_*t*_/*C*_0_) as a function of irradiation time (C), degradation of MB by the nanoclay@TiO_2_@PNVP hydrogel nanocomposite as a function of pH (D) and, plot of ln(*C*_*t*_/*C*_0_) as a function of pH (E).

Photocatalytic degradation activities of the synthesized hydrogel and hydrogel nanocomposites were evaluated with UV spectrophotometer measurements. Figure 10A displays the degradation of MB by the nanoclay@TiO_2_@PNVP hydrogel nanocomposite at 664 nm as a function of UV exposition time. The decrease in absorbance was associated with MB degradation. Figure 10B presents the degradation of MB as a function of irradiation time; when the mixed solutions were irradiated for 360 min, the degradation performance of the synthesized materials ranged from 0 to 98%. The PNVP hydrogel and nanoclay@PNVP hydrogel nanocomposites showed almost no photocatalytic activity, while TiO_2_-based hydrogel nanocomposites showed good photocatalytic performances of approximately 93% for TiO_2_@PNVP and 98% for nanoclay@TiO_2_@PNVP hydrogel nanocomposites. This indicated that the photocatalytic performance was gained by the addition of TiO_2_, and the presence of nanoclay helped attract MB to the matrix where TiO_2_ is located Figure 10B–E.

To evaluate the effect of solution pH the photocatalysis degradation, photocatalytic experiments were conducted using MB and the nanoclay@TiO_2_@PNVP hydrogel nanocomposite at different pH values (2–10). The results are given in Figure 10D. The highest adsorption and photocatalytic removal efficiencies of the nanoclay@TiO_2_@PNVP hydrogel nanocomposite material were obtained at pH 10, and 100% of MB was degraded within 150 min of UV irradiation. The photocatalytic degradation MB takes place on the surface of the nanoclay@TiO_2_@PNVP hydrogel nanocomposite. Therefore, as the amount of adsorbed material on the surface increases, the photocatalytic efficiency increases.

#### 3.5.1 Kinetics of Photodegradation of MB

**Figure 11:**
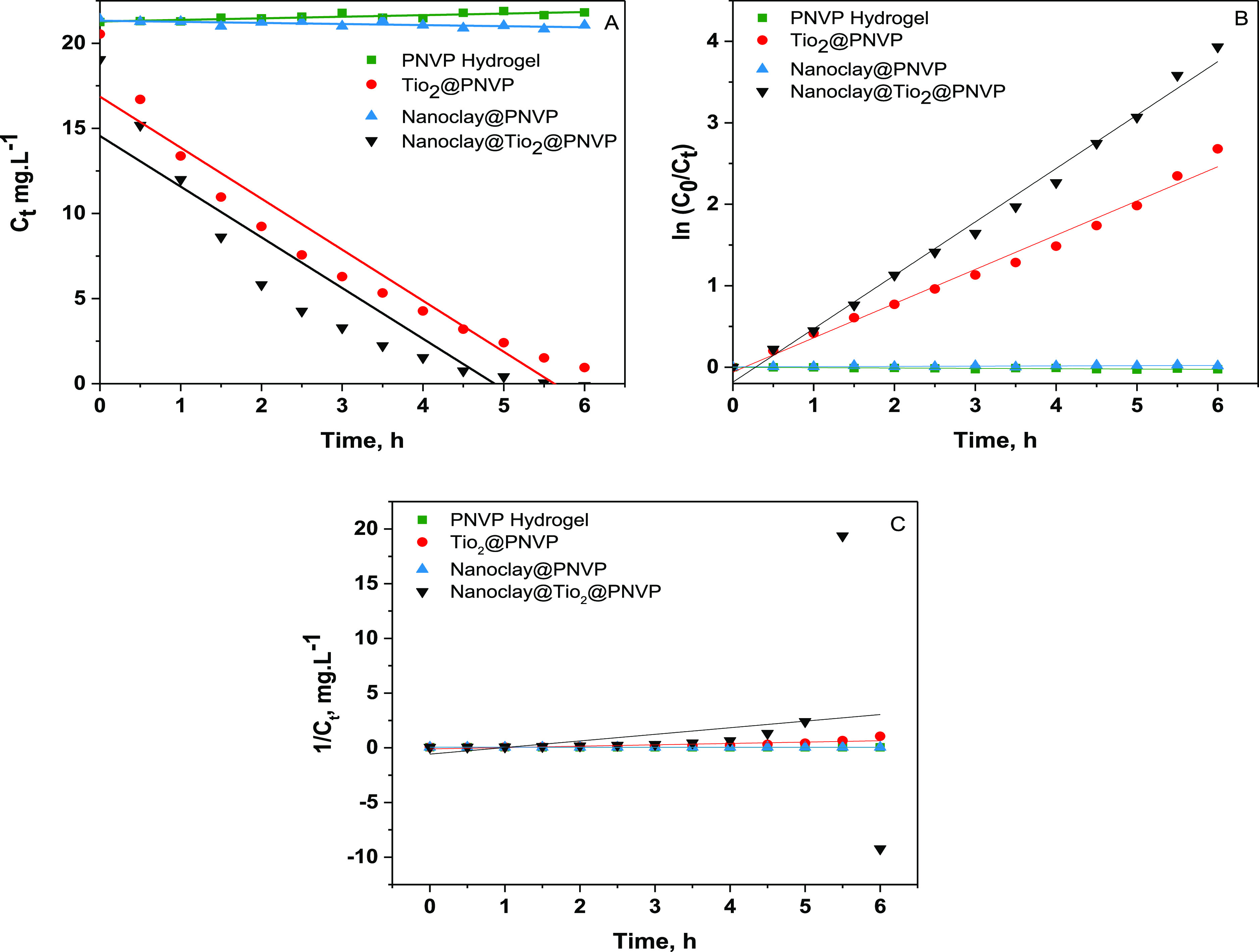
Kinetic plot for MB photodegradation. (A) Zero-order model, (B) first-order model, and (C) second-order model.

Figure 11 represents the kinetics model fitted for the pseudo-zero-order, first-order, and second-order models for MB photodegradation using the synthesized materials. Table 4 summarizes the rate constant *k* and its correlation coefficients (*R*^2^). The pseudo-first-order model with a correlation coefficient (*R*^2^) of 0.99 gave the best fit compared to the zero-order and second-order models. Therefore, the removal rate of MB using the synthesized materials follows the first-order kinetic model (Figure 11).

**Table 4:** Photodegradation Kinetics Parameters of MB

	zero-order		pseudo-first-order		pseudo-second-order	
sample	*k*′ (mg·g^–1^ h^–1^)	*R*^2^	*k*″ (mg·g^–1^ h^–1^)	*R*^2^	*k*‴ (mg·g^–1^ h^–1^)	*R*^2^
TiO_2_@PNVP	3	0.91	4.2 × 10^–3^	0.99	6	0.03
nanoclay@TiO_2_@PNVP	3	0.84	0.7	0.99	8	0.06

#### 3.5.2 Mechanism of Photodegradation of MB

**Figure 12:**
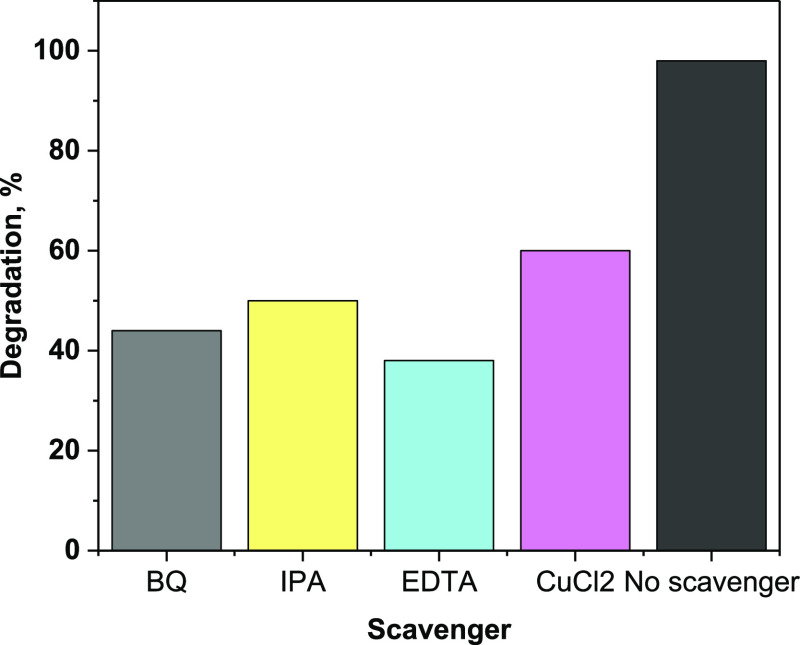
MB photodegradation in the presence of scavengers.

In a homogenous aqueous medium, generally, a photocatalyst under UV irradiation generates four highly reactive spices, holes (h^+^), superoxide (^•^O_2_^–^), electron (e^–^), and hydroxyl radicals (^•^OH); these spices participate in the photodegradation of the targeted organic molecule. Trapping experiments are performed to elucidate the roles of the major active oxidation spices in MB photodegradation by the nanoclay@TiO_2_@PNVP hydrogel nanocomposite. Therefore, EDTA was used as an h^+^ scavenger, IPA as ^•^OH, BQ as ^•^O_2_^–^, and CuCl_2_ as an e^–^ scavenger. The results of the experiments are presented in Figure 12. The presence of scavengers decreased the degradation percentage to 38% in presence of EDTA, 42% within BQ, 45% with IPA, and 60% in presence of CuCl_2_, indicating that all spices contribute to the photocatalytic process and that the h^+^ is the major generated spices. Furthermore, the final photodegraded intermediate was identified by GC–MS. The obtained GC–MS results proved that MB and SLD were completely converted to H_2_O and CO_2_.

The photodegradation mechanism of MB has been widely discussed in the literature, and it can be presented in steps.^44−46^ In the first step, the combination of high-water adsorption properties of the hydrogel and nanoclay helps the substances (H_2_O, MB) approach the surface of TiO_2_. Moreover, when the medium is irradiated with UV, the promotion of photoelectron from the filled valence band to the empty conduction band of TiO_2_ occurs, which generates electron and hole pair (e^–^/h^+^) (eq 18). In the second step, the photogenerated h^+^(VB) and (e_CB_^–^) react either with MB (oxidation and reduction reactions) or with water and the adsorbed oxygen at the surface of the semiconductor to produce hydroxyl (OH^•^) and anionic superoxide (O_2_^•–^) radicals, respectively (eqs 19 and 20). The anionic superoxide (O_2_^•–^) gets protonated to form hydroperoxyl radical (HOO^•^) the latest combine to form hydrogen peroxide (H_2_O_2_) which dissociates into highly reactive hydroxyl radicals (OH^•^) (eqs 21–23).

The generated spices are extremely reactive. They attack adsorbed organic molecules or those that are very close to the TiO_2_ surface causing their mineralization to H_2_O and CO_2_ (eqs 24–26).
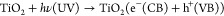
18
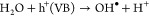
19
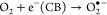
20
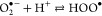
21
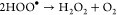
22
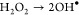
23
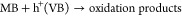
24
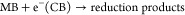
25
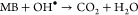
26

### 3.6 Reusability of the Nanoclay@TiO_2_@PNVP Hydrogel Nanocomposite

**Figure 13:**
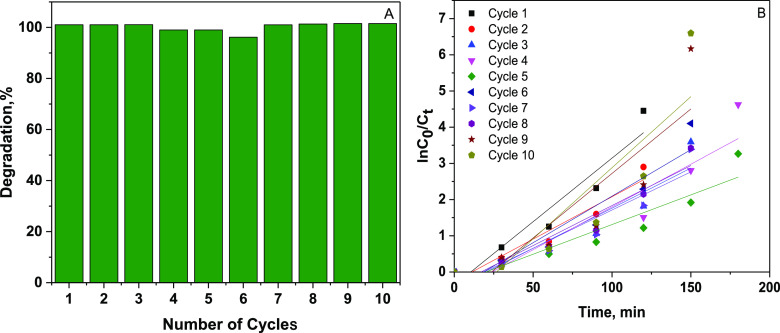
Reusability cycles of the nanoclay@TiO_2_@PNVP hydrogel nanocomposite for MB photodegradation process (A) and the plot of ln(*C*_*t*_/*C*_0_) as a function of cycle (B).

From an economic perspective, hydrogel regeneration is crucial. Therefore, the reusability of the nanoclay@TiO_2_@PNVP hydrogel nanocomposite was tested for both adsorption-based removal and photocatalytic degradation-based removal of MB at optimal conditions. Figure 13 displays 10 photocatalysis cycles, and the degradation percentages of MB over the 10 cycles were higher than 96%, demonstrating that the nanoclay@TiO_2_@PNVP hydrogel nanocomposite had a good recycling performance and stability for photocatalytic degradation of MB.

**Figure 14:**
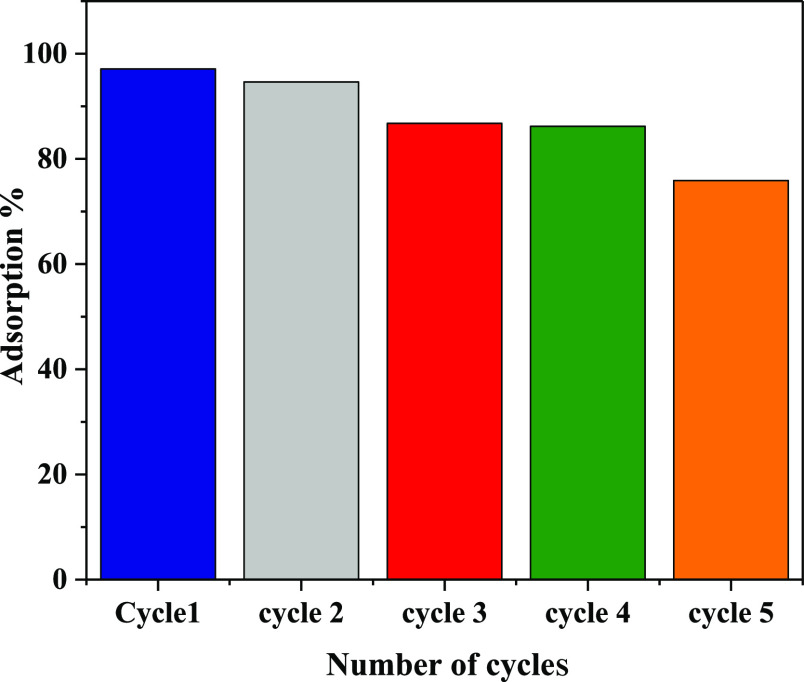
Reusability cycles of the nanoclay@TiO_2_@PNVP hydrogel nanocomposite for adsorption-based removal of MB.

Figure 14 shows five cycles of adsorption-based MB removal. In the first four cycles, the adsorption efficiencies were higher than 87%, and in the 5th cycle, the adsorption efficiency decreased to 75%. These results demonstrated that the nanoclay@TiO_2_@PNVP hydrogel nanocomposite could be used as an adsorbent for MB several times.

### 3.7 Adsorption-Based Removal of SLD

**Figure 15:**
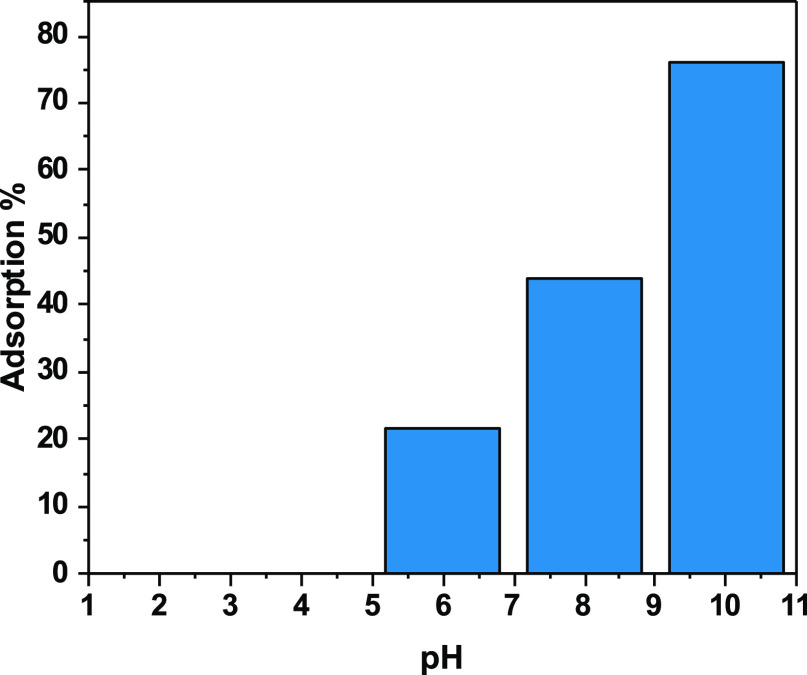
Adsorption of SLD as a function of pH variation using nanoclay@TiO_2_@PNVP as the adsorbent.

Figure 15 shows the variation of SLD adsorption percentage as a function of pH using the nanoclay@TiO_2_@PNVP hydrogel nanocomposite. From pH 2 to pH 4, there was no adsorption of SLD. With increasing the pH from 6 to 10, the adsorption percentage increases from 20% at pH 6 to attain a maximum of adsorption of 78% at pH 10. These variations were associated with the electrostatic interactions between SLD and the adsorbent. SLD is present in ampholyte form in the solutions. It combines moderate basicity and weak acidity character, so the acidity of the medium plays an important role in its sequestration. In mediums with pH below 6, both SLD and the adsorbent show a positive charge, which results in repulsion and low adsorption percentage. At upper pH values, the attraction between the adsorbent and SLD occurs because they own different charges, so the adsorption percentage increases.

### 3.8 Photocatalytic Degradation-Based Removal of SLD

**Figure 16:**
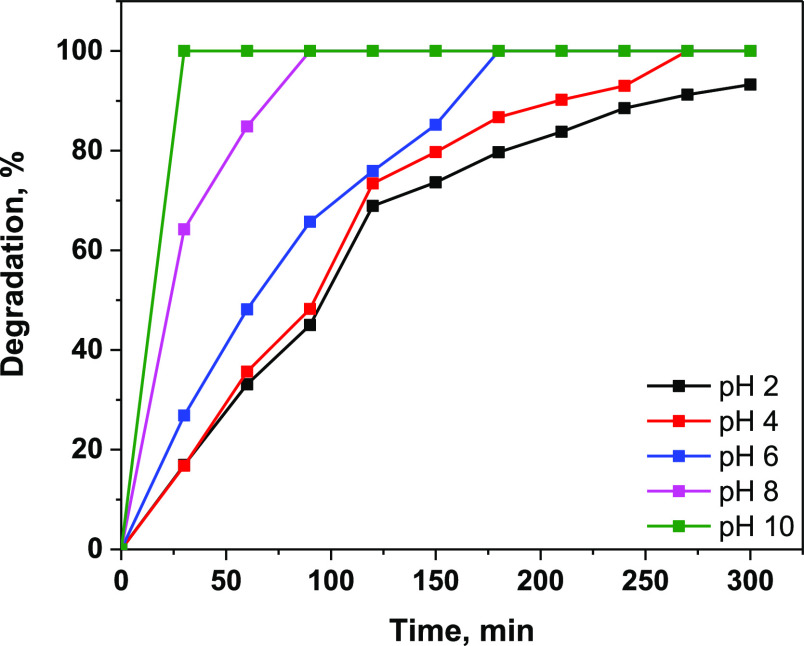
Photodegradation of SLD as a function of pH on nanoclay@TiO_2_@PNVP.

The photocatalysis of SLD using the nanoclay@TiO_2_@PNVP hydrogel nanocomposite at different pH values (2–10) was performed, and the results are given in Figure 16. The highest adsorption and photocatalytic removal efficiencies were obtained at pH 10, and 100% of SLD was degraded within 60 min of UV irradiation. The photocatalytic degradation takes place on the surface of the nanoclay@TiO_2_@PNVP hydrogel nanocomposite. Therefore, as the amount of adsorbed material on the surface increases, the photocatalytic efficiency increases.

Table 5 presents a summary of several works using hydrogel composites for the removal of organic molecules. The synthesized nanoclay@TiO_2_@PNVP was used for the adsorption and photocatalytic degradation of MB and SLD, and 20 ppm of MB and SLD were completely degraded within 150 and 60 min, respectively, which shows the enhanced adsorption and photocatalytic degradation of both SLD and MB.

**Table 5:** Summary of Several Works Using Hydrogel Composites for the Removal of Organic Dyes

		adsorption			photocatalysis			
photocatalyst/adsorbent	target molecule	amount of adsorbent (g)	adsorption percentage (%)	number of cycles	amount of photocatalyst (g)	removal efficiency (%)	number of cycles	refs
chitin-*cl*-poly(itaconic acid-co-acrylamide)/ZrW nanocomposite hydrogel	Sulphon Black	0.025	52.63	6	0.025	92.66	6	(47)
chitin-*cl*-poly(itaconic acid-*co*-acrylamide)/Fe_2_O_3_ composite hydrogel	Acid Orange 8	0.05	52		0.05	100		(48)
LNR-*g*-MaH/AAc/MMT	MB	0.55	99.28					(49)
PAM–rGO–P25 hydrogel	MB				0.23	100	5	(50)
cellulose/GO/TiO_2_ hydrogel	MB				5	93	10	(51)
nanoclay@TiO_2_@PNVP	MB	0.05	92	5	0.25	99	10	this work
	SLD		78			100		

## 4 Conclusions

In this study, the synthesis of the nanoclay@TiO_2_@PNVP hydrogel nanocomposite as a multifunctional material was successfully performed. The new material can be used in two treatment processes separately or in combination in one step, which provides an important advantage for its usability in environmental applications. The nanoclay@TiO_2_@PNVP hydrogel nanocomposite can be used as an adsorbent and photocatalyst of MB and SLD. It can be cleaned either in an acid solution or by a photocatalytic process, and it can be used at least 5 times as an adsorbent and 10 times as a photocatalyst for the removal of MB. The adsorption and photodegradation of MB were found to be pH-dependent, the adsorption efficiency was found as 98% at pH 10 and photocatalysis degradation at the same pH value. The incorporation of TiO_2_ NPs into the hydrogel led to a gain in photocatalytic performance resulting in 98% of MB degradation efficiency. This study dived slightly deeper into some environmentally required studies such as the simultaneous removal of organic pollutants from environmental samples. We believe that this new concept that we have developed has the potential to be applied to large-scale wastewater treatment systems in the future.
